# Impact of Childhood Nutritional Status on Pathogen Prevalence and Severity of Acute Diarrhea

**DOI:** 10.4269/ajtmh.17-0139

**Published:** 2017-08-21

**Authors:** Kirkby D. Tickell, Patricia B. Pavlinac, Grace C. John-Stewart, Donna M. Denno, Barbra A. Richardson, Jaqueline M. Naulikha, Ronald K. Kirera, Brett E. Swierczewski, Benson O. Singa, Judd L. Walson

**Affiliations:** 1 University of Washington, Seattle, Washington;; 2 The Childhood Acute Illness & Nutrition Network, Nairobi, Kenya;; 3 Kenya Medical Research Institute (KEMRI), Nairobi, Kenya;; 4 United States Army Medical Research Directorate–Kenya (USAMD-K), Nairobi, Kenya

## Abstract

Children with acute and chronic malnutrition are at increased risk of morbidity and mortality following a diarrheal episode. To compare diarrheal disease severity and pathogen prevalence among children with and without acute and chronic malnutrition, we conducted a cross-sectional study of human immunodeficiency virus-uninfected Kenyan children aged 6–59 months, who presented with acute diarrhea. Children underwent clinical and anthropometric assessments and provided stool for bacterial and protozoal pathogen detection. Clinical and microbiological features were compared using log binomial regression among children with and without wasting (mid-upper arm circumference ≤ 125 mm) or stunting (height-for-age *z* score ≤ −2). Among 1,363 children, 7.0% were wasted and 16.9% were stunted. After adjustment for potential confounders, children with wasting were more likely than nonwasted children to present with at least one Integrated Management of Childhood Illness danger sign (adjusted prevalence ratio [aPR]: 1.3, 95% confidence interval [CI]: 1.0 to 1.5, *P* = 0.05), severe dehydration (aPR: 2.4, 95% CI: 1.5 to 3.8, *P* < 0.01), and enteroaggregative *Escherichia coli* recovered from their stool (aPR: 1.8, 1.1–2.8, *P* = 0.02). There were no differences in the prevalence of other pathogens by wasting status after confounder adjustment. Stunting was not associated with clinical severity or the presence of specific pathogens. Wasted children with diarrhea presented with more severe disease than children without malnutrition which may be explained by a delay in care-seeking or diminished immune response to infection. Combating social determinants and host risk factors associated with severe disease, rather than specific pathogens, may reduce the disparities in poor diarrhea-associated outcomes experienced by malnourished children.

## INTRODUCTION

Two-thirds of the global childhood mortality attributed to diarrhea is associated with malnutrition, yet the mechanisms linking this critical risk factor to death are poorly understood.^1^ A child with severe acute malnutrition (SAM) is at up to 12 times higher risk of death due to diarrheal illness than better nourished children.^2–4^ Similarly, chronic malnutrition (stunting), or decreased height-for-age z score (HAZ), is another important prognostic indicator for death during and after a moderate-to-severe diarrhea episode.^5^ A better understanding of how these two risk factors promote adverse outcomes is needed to end childhood diarrheal mortality.

Host factors associated with malnutrition, such as a compromised immune system, environmental enteric dysfunction, and enteric microbiome dysbiosis may predispose malnourished children to more severe disease.^6–9^ Children with malnutrition may also be more likely to live in households of low socioeconomic status where poor access to clean water,^10,11^ sanitation, and hygiene may expose them to greater fecal microbial loads and higher risk of pathogens associated with mortality such as *Shigella* species (spp.), ST-enterotoxigenic *Escherichia coli* (ETEC), typical enteropathogenic *Escherichia coli* (*E. coli*, EPEC), and *Cryptosporidium*.^5^ Finally, children from households with lower socioeconomic status may present for care later during an episode of acute diarrhea, leading to more severe illness at presentation.^12^

We sought to explore the relationship between nutritional status and presence of enteropathogens, as well as disease severity, using data from a cross-sectional study of children recruited at presentation with acute diarrhea to health facilities in the Nyanza province of Kenya.

## MATERIALS AND METHODS

This analysis used data collected from children under 5 years of age presenting with acute diarrhea to the outpatient and inpatient units at Kisii Provincial Hospital, Migori District Hospital, and Homa Bay District Hospital between November 2011 and June 2014. A description of this study has been published previously.^13,14^ Briefly, the parent study recruited children aged 6 months to 15 years presenting to the health facility with acute diarrhea (≥ 3 loose stools in the previous 24 hours and lasting less than 14 days). Eligible participants were excluded if they were not accompanied by a legal guardian or biological parent, if study staff were unable to collect a stool sample or rectal swab or if the primary caregiver refused human immunodeficiency virus (HIV) testing on behalf of the child. Written informed consent was obtained from the child’s caregiver. For this analysis, we included HIV-uninfected children 6 months to 5 years of age recruited at Homa Bay or Kisii hospitals. Migori Hospital patients were excluded as the low numbers recruited would be challenging to appropriately include in statistical models. The study was approved by the Kenya Medical Research Institute and University of Washington ethical review boards.

At enrollment the primary caregiver completed a standardized clinical and sociodemographic questionnaire. All children were examined by clinical staff who recorded the presence of World Health Organization (WHO) Integrated Management of Childhood Illness (IMCI) danger signs (unable to drink or feed, vomiting everything, convulsions, lethargic, or unconscious) and hydration status (severe dehydration defined as at least two of the following: lethargic or unconscious, sunken eyes, not able to drink, reduced skin turgor). Weight, mid-upper arm circumference (MUAC), and height (or length) were measured and HAZs, weight-for-age z-scores (WAZ), and weight-for-height z-scores (WHZ) were calculated using WHO ANTHRO software.^15^ Z-scores of less than −7 or greater than 7 were deemed implausible and set to missing. All children were tested for HIV using antibody testing (Abbott Determine^™^ rapid test kit [Abbott Park, IL] and confirmed using Uni-Gold^™^ [Trinity Biotech, Bray, Ireland]) or HIV polymerase chain reaction (PCR) if under 18 months of age. Maternal HIV status was determined by antibody rapid testing or self-report. Malaria status was determined by a combination of rapid test (Paracheck Pf^®^ Orchid Biomedical Services, Verna, Goa, India) and microscopy.

Stool samples were collected in stool collection containers by caregivers or study staff. When a child was not able to produce stool, three rectal swabs were obtained. Fecal samples were collected prior to antibiotic administration, if indicated. Samples were received in the nearby U.S. Army Medical Research Directorate—Kenya (USAMD-K) Microbiology Hub in Kericho within 24 hours of collection after being placed in transport media (Cary–Blair for bacterial culture and 10% formalin for protozoal testing) and maintained at 2–8°C. Bacterial pathogens (*E. coli, Shigella* spp., *Campylobacter* spp., *Salmonella* spp.) were identified using traditional culture and serotyping methods confirmed using the MicroScan WalkAway 40 Plus (Beckman Coulter, Brea, CA) automated platform. *Escherichia coli* isolates were further tested for virulence factors using multiplex PCR and classified by pathotype: ETEC (heat labile or heat stable enterotoxin), EPEC (bundle forming pilus and, after March 2013, intimin), enteroaggregrative *E.coli* (EAEC) (*aatA* and, after March 2013, *aaiC*), enteroinvasive *E.coli* (invasion plasmid antigen H), or enterohemmorhagic *E.coli* (Shiga toxin 1,2 and variant). Protozoal infections were identified from whole stool samples using microscopy after stool concentration using Mini Parasep^®^ Solvent Free concentration kit (DiaSys, Berkshire, England). No viral testing was performed.

### Statistical analysis.

Analyses were conducted to examine whether children with acute malnutrition (wasting, MUAC < 12.5 cm) or chronic malnutrition (stunted HAZ < −2) had a higher prevalence of detectable enteric pathogens at presentation or presented with more severe disease (defined as the presence of at least one IMCI danger sign and/or severe dehydration). WHZ was not used to define acute malnutrition because it is influenced by fluid status and children with diarrhea often have some degree of dehydration. All prevalences were compared using log-binomial regression and associated χ^2^ tests.^16^ Pathogens and danger signs that were significantly associated with nutritional status in univariate regression at an alpha of 0.05 were further assessed in multivariable models. Potential confounders were evaluated for inclusion in each model in a stepwise manner and maintained in the model if the prevalence ratio changed by more than 10%. The following potential confounding variables were considered: age, gender, hospital site, currently receiving any breast milk (yes/no), maternal HIV status, family income (dichotomized at above or below 5,000 Kenyan Shillings), caregiver education (primary education or less, some secondary education, or secondary or greater educational level), persons per room, and access to improved sanitation (as defined by WHO/United Nations Children's Fund Joint Monitoring Program).^17^ Finally, the linearity of significant univariate associations with acute malnutrition were examined graphically by further categorizing MUACs into: < 11.5 (SAM), ≥ 11.5 to < 12.5 (moderate acute malnutrition), ≥ 12.5 to < 13.5, ≥ 13.5 to < 14.5 and ≥ 14.5. No similar descriptive analysis was done for stunting as the initial results were not significant. All analyzes were conducted using Stata 13.1 (Statacorp; College Station, TX).

## RESULTS

The parent study enrolled 1,758 children. Children over the age of five (213, 12.1%), those recruited at Migori hospital (81, 4.6%), those with HIV infection (76, 4.3%) and those with unknown HIV status (25, 1.4%) were excluded from this analysis. The median age of the remaining 1,363 children included in this analysis was 20 months (interquartile range: 11–36 months). Eighty-nine (6.5%) of the included children were recruited from the hospital’s inpatient services. All children had MUAC data available, although 36 (2.6%) children did not have height/length recorded. Fifty-seven (4.2%) children were classified as having acute malnutrition without being stunted, 194 (14.2%) were stunted without acute malnutrition and 35 (2.6%) were both acutely malnourished and stunted.

### Acute malnutrition.

Children with acute malnutrition were younger (mean age: 12.7 versus 24.7 months, *P* < 0.01, Table 1), had more frequent reported antibiotic use in the last 7 days (19.6% versus 11.9%, *P* = 0.04), more likely to come from Homa Bay county (63.9% versus 50%, *P* < 0.01) and had a higher prevalence of stunting (38.0% versus 15.4%, *P* < 0.01) than children without acute malnutrition. Caregivers of children with acute malnutrition were more likely to report household income < 5,000 Kenyan shillings per-month (KSH, 57.7% versus 36.6%, *P* < 0.01) and lack of access to improved sanitation (11.4% versus 2.7%, *P* < 0.01) than caregivers without acute malnutrition. In addition, caregivers of acutely malnourished children reported lower educational achievement (greater than primary school education: 23.5 versus 52.8, *P* < 0.01) and a higher prevalence of HIV (27.0% versus 9.4%, *P* < 0.01). Finally, acutely malnourished children were more likely to have traveled for longer than 1 hour to reach the health facility (33.0% versus 15.9%, *P* < 0.01) and were more likely to have previously sought medical care for the current episode of illness (39.6% versus 28.0%, *P* = 0.02).

**Table 1:** Demographic and clinical differences at diarrhea presentation among children with MAM or SAM compared with children without either condition

	Acute Malnutrition (MUAC < 12.5 cm) *N* = 97*	No MAM or SAM (MUAC ≥ 12.5 cm) *N* = 1,293
	*N*/mean (%/SD)	*N*/mean (%/SD)
Child		
Hospital of presentation		
Homa Bay	62 (8.7)	647 (91.3§)
Kisii	35 (5.1)	646 (94.9§)
Inpatient recruitment‡	24 (25.0)	65 (5.3§)
Age in months	12.7 (7.9)	24.7 (15.1§)
Sex (male)	50 (51.6)	692 (53.5)
Currently breast feeding (if < 24 months)	63 (71.6)	541 (78.4)
Months exclusively breastfed	4.6 (1.7)	5.2 (1.9§)
Antibiotics in last 7 days (reported)	19 (19.6)	154 (11.9§)
Stunted (HAZ < −2)	35 (38.0)	194 (15.4§)
Blood in stool (parent/caretaker reported)	0 (0.0)	17 (1.3)
Malaria RDT+	12 (12.4)	130 (10.1)
Rectal swab used	11 (11.3)	93 (7.2)
Caregiver		
Biological mother is primary caregiver	91 (93.8)	1,209 (93.5)
Caregiver education		
Primary or less	62 (76.5)	525 (47.2§)
Some secondary	13 (16.1)	346 (31.1§)
Greater than secondary	6 (7.4)	241 (21.7§)
Caregiver HIV infected†	24 (27.0)	112 (9.4§)
Socioeconomic status		
Income < 5,000 KSH	56 (57.7)	473 (36.6§)
Persons per room in house	2.6 (1.2)	2.3 (1.3§)
Improved water source	74 (76.3)	1,067 (82.8)
Improved toilet	86 (88.6)	12.57 (97.3§)
Care seeking		
Previously sought care for this illness	38 (39.6)	345 (28.0§)
Consulted a traditional healer	2 (2.1)	18 (1.4)
> 1 hour travel time to clinic	32 (33.0)	206 (15.9§)

HAZ = height-for-age z score; HIV = human immunodeficiency virus; KSH = Kenyan Shillings; MAM = moderate acute malnutrition; MUAC = mid-upper arm circumference; RDT = rapid diagnostic test; SAM = severe acute malnutrition; SD = standard deviation.

* Thirty-two children had SAM, 65 MAM.

† Eight (8.3%) caregivers of children with acute malnutrition and 99 (7.7%) caregivers of children without SAM/MAM had an unknown HIV status and declined testing.

‡ Inpatient recruitment indicates how many children were recruited from the inpatient ward, but recruitment in the outpatient department does not exclude subsequent admission.

§ Significant differences (*P* < 0.05) between acutely malnourish and better nourished children were observed, a two-sided fisher’s exact test was used for categorical variables, a *t* test was used for continuous variables. Disaggregate values for SAM, MAM are given in the Supplemental Appendix Table 1.

**Figure 1.**
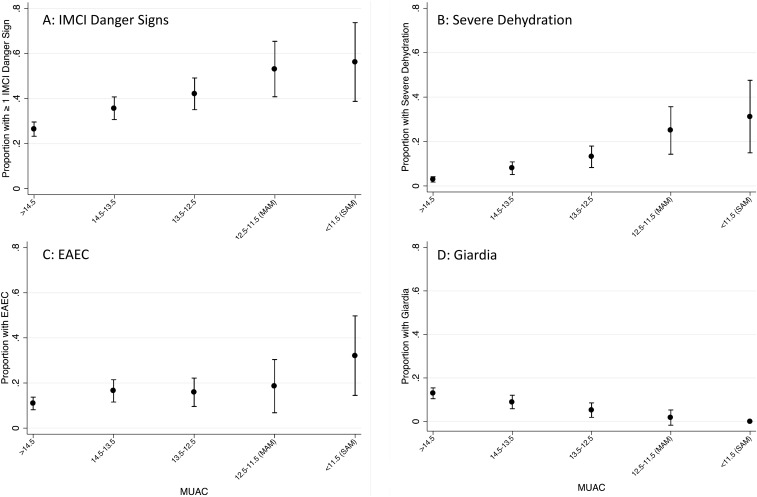
(**A**) Presence of at least one Integrated Management of Childhood Illness (IMCI) danger sign across mid-upper arm circumference (MUAC) categories. (**B**) Severe dehydration at presentation across MUAC categories. (**C**) The trend in enteroaggregative *Escherichia coli* (EAEC) prevalence across MUAC categories. (**D**) The trend in *Giardia* prevalence across MUAC categories.

Children with acute malnutrition were significantly more likely to present with severe illness. After adjusting for child age and caregiver education, children with low MUAC were 2.4 times more likely to present with severe dehydration (adjusted prevalence ratio [aPR]: 2.4, 95% confidence interval [CI]: 1.5–3.8, *P* < 0.01, Table 2) than their better nourished counterparts, and were also more likely to present with at least one IMCI danger sign after adjusting for age and site (aPR: 1.3, 95% CI: 1.0 to 1.5, *P* = 0.05). There was an incremental relationship between MUAC and disease severity, such that even among MUAC categories traditionally considered to be normal, the prevalence of severe dehydration or at least one IMCI danger sign increased with decreasing in MUAC (Figure 1A and B).

**Table 2:** Enteric pathogens and severity of diarrhea in children with and without acute malnutrition

Infection*	Acute Malnutrition (MUAC < 12.5 cm) *N* = 97	No MAM or SAM (MUAC ≥ 12.5 cm) *N* = 1,293	Unadjusted prevalence ratio (95% CI)	Adjusted prevalence ratio (95% CI)
	*N* (%)	*N* (%)		
Bacteria				
*Campylobacter* spp.	5 (5.2)	96 (7.4)	0.7 (0.3–1.7)	
EAEC†	17 (23.9)	110 (13.2)	2.0 (1.3–3.2)	1.8 (1.1–2.8)‡
EIEC†	2 (2.8)	25 (3.0)	0.9 (0.2–3.9)	
EHEC†	0 (0.0)	2 (0.2)	–	
EPEC-atypical†	1 (1.9)	18 (2.3)	0.8 (0.1–6.1)	
EPEC-typical†	4 (5.6)	32 (3.8)	1.5 (0.5–4.0)	
ETEC†	2 (2.8)	35 (4.2)	0.7 (0.2–2.7)	
*Salmonella* spp.	2 (2.1)	16 (1.2)	1.7 (0.4–7.1)	
*Shigella* spp.	4 (4.1)	61 (4.7)	0.9 (0.3–2.4)	
Protozoa				
*Giardia* spp.	1 (1.2)	129 (10.8)	0.1 (0.0–0.9)	0.2 (0.0–1.3)§
*Cryptosporidium* spp.	3 (3.5)	51 (4.3)	0.8 (0.3–2.6)	
*Entaeombea* spp.	1 (1.2)	21 (1.8)	0.7 (0.1–4.8)	
Severity				
≥ 1 danger sign	52 (54.2)	402 (31.3)	1.7 (1.4–2.1)	1.3 (1.0–1.5)¶
Severe dehydration	26 (27.1)	75 (5.8)	4.6 (3.1–6.9)	2.4 (1.5–3.8) §

CI = confidence interval; EAEC = enteroaggregative *Escherichia coli*; EIEC = enteroinvasive *E.coli*; EHEC = enterohemmorhagic *E.coli*; EPEC = enteropathogenic *E.coli*; ETEC = enterotoxigenic *Escherichiacoli*; MAM = moderate acute malnutrition; MUAC = mid-upper arm circumference; MUAC = mid-upper arm circumference; SAM = severe acute malnutrition.

* No pathogen was identified 52 (60.5%) children with acute malnutrition and 716 (59.7%) children without acute malnutrition.

† Two *E. coli* serotypes were identified in 2 (2.1%) children with acute malnutrition and 23 (1.8%) children without MAM/SAM. Only 905 samples had *E.coli* serotyping performing (103 with acute malnutrition, 802 without SAM/MAM), of these children 827 were tested for atypical EPEC.

‡ Adjusted for age, caregiver education, person per room (toilet type was dropped from the model due to colinearity with caregiver education).

§ Adjusted for age and caregiver education.

¶ Adjusted for age and center. Disaggregated values for SAM, MAM are given in the Supplemental Appendix Table 2.

Significant differences in pathogen detection were noted across MUAC categories (Table 2). Children with acute malnutrition were 1.8 times more likely to have EAEC detected in their stools than children without wasting in the adjusted model (95% CI: 1.1 to 2.8, *P* = 0.02). EAEC prevalence also tracked, inversely, with MUAC categories (Figure 1C). Conversely, *Giardia* infections were significantly more common in stools of children without acute malnutrition in the crude models (PR 0.1, 95% CI: 0.0 to 0.9, *P* = 0.02), although this association was attenuated by adjustment for age and caregiver education (aPR 0.2, 95% CI: 0.0 to 1.3, *P* = 0.08, Figure 1D). No differences in prevalence of other pathogens between children with and without acute malnutrition were observed.

### Chronic malnutrition.

When compared with children with HAZs greater than −2, stunted children were more likely to be male (62.5% versus 51.4%, *P* < 0.01, Table 3), acutely malnourished (38.0% versus 15.4%, *P* < 0.01) and have an HIV infected primary caregiver (17.5% versus 9.6%, *P* < 0.01). The households of stunted children were also more likely to have a low income (< 5,000 KSH/month: 49.6% versus 37.9%, *P* < 0.01).

**Table 3:** Demographic and clinical factors among children with and without stunting (chronic malnutrition) presenting with acute diarrhea

	Stunted (HAZ ≤ −2) *n* = 229	Not Stunted (HAZ > −2) *n* = 1,126
	*N*/mean (%/SD)	*N*/mean (%/SD)
Child		
Hospital of presentation		
Homa Bay	119 (17.0)	583 (83.1)
Kisii	110 (16.9)	543 (83.2)
Inpatient recruitment*	24 (10.7)	59 (5.5§)
Age in months	21.7 (13.2)	24.4 (15.4§)
Sex (male)	143 (62.5)	579 (51.4§)
Currently breast feeding (if < 24 months)	103 (72.0)	478 (78.5)
Months exclusively breastfed	5.1 (1.9)	5.1 (1.9)
Antibiotics in last 7 days (reported)	30 (13.1)	138 (12.3)
Wasted (MUAC < 12.5 cm)	35 (38.0)	194 (15.4§)
Blood in stool	0 (0.0)	16 (1.4)
Malaria RDT+	25 (10.9)	115 (10.2)
Rectal swab used	14 (6.1)	87 (7.7)
Caregiver		
Biological mother is caregiver	213 (93.0)	1,053 (93.5)
Caregiver education		
Primary or less	113 (58.9)	459 (47.2§)
Some secondary	49 (25.5)	302 (31.1§)
Greater than secondary	30 (15.6)	211 (21.7§)
Caregiver HIV+†	37 (17.5)	99 (9.6§)
Socioeconomic status		
Income < 5,000 KSH	109 (47.6)	404 (35.9§)
Persons per room in house	2.4 (1.3)	2.3 (1.3)
Improved water source	196 (85.6)	919 (81.9)
Unimproved toilet	220 (96.1)	1,088 (96.7)
Care seeking		
Previously sought care for this illness	64 (28.4)	306 (28.6)
Consulted a traditional healer	0 (0.0)	20 (1.8§)
> 1 hour travel time to clinic	49 (21.4)	180 (16.0§)

HIV = human immunodeficiency virus; MUAC = mid-upper arm circumference; RDT = rapid diagnostic test; SD = Standard deviation.

* Inpatient recruitment indicates how many children were recruited from the inpatient ward, but recruitment in the outpatient department does not exclude subsequent admission.

† Seventeen (7.4%) caregivers of children with stunting and 89 (7.9%) caregivers of children without stunting had an unknown HIV status and declined testing.

‡ Significant difference between stunted and better nourished children, a fisher’s exact test was used for categorical variables, *t* test was used for continuous variable.

In the categorical analysis of HAZ, we found no significant differences in the prevalences of severe dehydration or IMCI danger signs between stunted and nonstunted children (Table 4). Similarly, no differences in enteric pathogen prevalence were observed between groups of children stratified by stunting status.

**Table 4:** Enteric pathogens and severity of diarrhea in children with and without stunting

Infection*	Stunted (HAZ < −2) *N* = 229	Not stunted (HAZ > −2) *N* = 1,126	Unadjusted prevalence ratio (95% CI)	Adjusted prevalence ratio (95% CI)
	*N* (%)	*N* (%)		
Bacteria				
*Campylobacter* spp.	22 (9.6)	78 (6.9)	1.4 (0.9 to 2.2)	–
EAEC†	23 (14.5)	100 (13.8)	1.0 (0.7 to 1.6)	–
EIEC†	4 (2.5)	23 (3.2)	0.8 (0.3 to 2.3)	–
EHEC†	0 (0.0)	2 (0.3)	–	–
EPEC-atypical†	4 (2.8)	15 (2.3)	1.2 (0.4 to 3.7)	–
EPEC-typical†	6 (3.8)	29(4.0)	0.9 (0.4 to 2.2)	–
ETEC†	8 (5.0)	28 (3.9)	1.3 (0.6 to 2.8)	–
*Salmonella* spp.	4 (1.8)	14 (1.2)	1.4 (0.5 to 4.2)	–
*Shigella* spp.	9 (3.9)	55 (4.9)	0.8 (0.4 to 1.6)	–
Protozoa				
*Giardia* spp.	21 (9.8)	107 (10.3)	0.9 (0.6 to 1.5)	–
*Cryptosporidium* spp.	6 (2.8)	47 (4.5)	0.6 (0.3 to 1.4)	–
*Entaeombea* spp.	6 (2.8)	16 (1.6)	1.8 (0.7 to 4.6)	–
Severity				
≥ 1 danger sign	81 (35.7)	359 (32.1)	1.1 (0.9 to 1.4)	–
Severe dehydration	15 (6.6)	81 (7.2)	0.9 (0.5 to 1.6)	–

CI = confidence interval; EAEC = enteroaggregative *Escherichia coli*; EIEC = enteroinvasive *E.coli*; EHEC = enterohemmorhagic *E.coli*; EPEC = enteropathogenic *E.coli*; ETEC = enterotoxigenic *Escherichiacoli*; HAZ = height-for-age z score; MUAC = mid-upper arm circumference.

* No pathogens were identified in 119 (55.4%) children with stunting and 626 (60.3%) children without stunting.

† Two *E.coli* serotypes were identified in three (1.3%) children with stunting and 21 (1.9%) children without stunting. Only 906 included children had *E.coli* serotyping performing (151 stunted children, 735 children without stunting), of these children 622 were tested for atypical EPEC.

## DISCUSSION

In this analysis, children with a MUAC less than 12.5 cm presenting with acute diarrhea were more likely to have severe disease than their better nourished peers. Other than a modestly increased prevalence of EAEC among wasted children, we did not identify specific pathogens associated with nutritional status including enteric pathogens previously shown to be strongly associated with mortality following an episode of moderate-to-severe diarrhea (*Shigella* spp., ST-ETEC, typical EPEC, or *Cryptosporidium*).^3,5^ This suggests that the observed increase in disease severity among wasted children was not due to a higher prevalence of virulent pathogens. Increased severity of diarrhea among malnourished children has previously been observed in a study of children living in rural Bangladesh.^19^ Two previous studies that examined diarrheal pathogen prevalence across nutritional strata found one or more of *Cryptosporidium*, EAEC, ETEC, and *Entamoeba histolytica* to be crudely associated with acute malnutrition, and a third study found a composite of all bacterial pathogens, including virulent *E.coli*, *Shigella* spp, *Salmonella* spp, *Campylobacter* spp, and *Plesiomonas shigelloides,* to also have a crude association with acute malnutrition.^20–22^ However, these studies also noted age to be associated with both pathogens and with malnutrition, but did not present age-adjusted models.

The severity of other infectious syndromes, including malaria and pneumonia, have been associated with acute malnutrition.^23–25^ A study of children presenting to Kilifi Hospital (Kenya), found that children with acute malnutrition were twice as likely to be bacteremic, and five times more likely to have *E. coli* bacteremia, than nonmalnourished children.^26^ This result was not explained by confounding due to age or HIV status. Poor intestinal barrier function may be an important cause of both this increased risk of bacteremia and the increased risk of death during a diarrheal episode. Intestinal permeability is a cardinal pathophysiologic process in environmental enteric dysfunction, a disease process that is thought to be a common cause of suboptimal growth, and has been described in children with kwashiorkor.^8,27^ This gut “leakiness” may permit pathogens to translocate into the systemic circulation which may explain the increased incidence of gram-negative bacteremia observed in the Kilifi study, and the increased risk of death during diarrheal episodes observed in other studies. Aside from facilitating overt bacteremia, translocation of microbial products such as bacterial antigens may provoke systemic inflammation that could diminish the immune response to challenges.^7,8^ Such an immune dysfunction may explain why malnourished children have more severe disease and a worse prognosis than better nourished peers, even when both groups are infected by similar pathogens. However, colonization with gram-negative bacteria in other organ systems, such as the upper and lower respiratory tract, is relatively common among children presenting to hospitals in low-resource settings,^23,28,29^ and may be another source of invasive infections.

In addition to possible pathophysiological mechanisms, social factors may underpin the association between acute malnutrition and diarrhea severity. For example, malnourished children are likely to present to health-care facilities later in their diarrheal episode.^12^ Delayed care seeking may be driven by multiple factors associated with malnutrition, including limited financial resources, difficulty accessing clinical facilities, or a distrust of health-care services.^12^ Indeed, our data suggest that children with acute malnutrition typically came from lower income households and often traveled for over an hour to reach care, reinforcing the hypothesis that financial and geographic barriers contribute to late presentation and increased disease severity.

The increased disease severity observed in children with acute malnutrition appears to be independent of pathogen and may be a function of impaired host response to infection or to late presentation to care, as discussed earlier. Interestingly, we did observe a higher prevalence of EAEC, a pathogen which has been associated with acute diarrhea in previous studies.^30,31^ Several other studies have observed associations between asymptomatic EAEC carriage and malnutrition, and between EAEC and persistent diarrhea.^32–34^ Long-term EAEC carriage may cause malnutrition by leading to enteric inflammation and subsequent malabsorption or systemic inflammation.^35^ One of the few previous diarrhea studies to compare enteric pathogens between nutritional strata found EAEC coinfection was significantly more common among malnourished children.^21^ It may be that EAEC is carried by malnourished children for extended periods of time but is only tested for and detected when another infection causes diarrhea. Alternatively, host vulnerability or variations in EAEC subtype could influence pathogenicity. For example, EAEC is considered an important cause of acute diarrhea in select populations, such as immunocompromised individuals, and a recent review noted that the considerable genetic diversity of EAEC strains may explain their variable propensity to cause symptomatic disease.^30,35,36^ The immunological deficits associated with malnutrition may allow EAEC infection to cause acute diarrhea in these vulnerable children.

In this analysis, children with acute malnutrition had a lower prevalence of *Giardia* recovery in their stools than better nourished children, although it should also be noted that accurate adjustment for age and socioeconomic status was not possible, as only a single child with acute malnutrition had *Giardia*. This result is at odds with previous studies that have found *Giardia* to be more or equally prevalent in stools of children with poor nutritional status.^37^ The role of *Giardia* as a cause of diarrhea is also unclear. Nonrandomized experimental studies in high-income countries have observed an association between *Giardia* infection and diarrhea,^38–40^ and its role as a diarrheal pathogen has been reinforced by localized diarrhea epidemics attributed to the parasite across Europe and the United States. However, multiple case–control studies from low-income settings have recovered *Giardia* more frequently in asymptomatic controls than diarrhea cases.^5^ These studies, coupled with in vitro evidence, suggest that *Giardia* may protect against severe diarrhea by modulating the pro-inflammatory response to coinfection with another pathogen.^38–40^ If this hypothesis is true, the higher prevalence of *Giardia* among better nourished could moderate the response to other enteric pathogens, leading to a decreased severity diarrhea when compared with children with less prevalent *Giardia*. Alternatively, antibiotic use, specifically with metronidazole, could have influenced these results. We observed an inverse relationship between MUAC and reported antibiotic use prior to presentation in our study. A recent publication reporting findings from 2,089 children in eight countries noted that metronidazole use in response to diarrheal episodes accounted for a substantial amount of the observed inverse association between *Giardia* and diarrhea.^41^

No significant relationship between stunting status and pathogen detection or disease severity was observed in this analysis. Among children with moderate-to-severe diarrhea in the largest study to address this topic (GEMS), HAZ was inversely associated with mortality.^5^ Previous studies have also shown the IMCI danger signs and classification of severe dehydration to be associated with a higher risk of death.^19^ In this study, we did not see an association between being stunted and the IMCI general danger signs or dehydration, which implies, if stunting is linked with poor diarrheal outcomes, that stunting may be associated with mortality through a pathway not being identified by the IMCI algorithm. GEMS found that two-thirds of deaths in the 90-days after moderate-to-severe diarrhea presentation occur more than 1 week after the initial diarrheal episode had begun.^5^ Stunting associated diarrheal deaths may occur in this late mortality period, while the IMCI danger signs and the classification of severe dehydration may indicate acute risk. This may suggest that becoming stunted and then developing diarrhea is indicative of generally deteriorating health, and it may be that this downward trajectory leads to poor outcomes rather than the severity of the acute diarrheal episode.

This analysis has multiple strengths, including utilizing data from a large sample of children over a wide range of nutritional statuses. However, this study was cross sectional and these data do not allow us to test whether differences in pathogen distribution or severity at presentation mediate the relationship between nutritional status and death, as mortality data were not collected. We are also unable to determine the temporal relationship between pathogen presence and malnutrition. Another limitation of this analysis is the reliance on a measured MUAC that was obtained during an episode of acute diarrhea. Despite being more resilient to dehydration than weight, MUAC may still be decreased during states of severe dehydration. A study in Bangladesh suggested that a state of 10% dehydration correlated with only a 0.03 cm decrease in MUAC,^42^ but another study in Kenya found a 20% overdiagnosis of SAM when using MUAC among dehydrated children.^43^ Both studies found MUAC to be more resistant to this misclassification than weight. Therefore, when assessments of postrehydration weight are not available, MUAC is currently the best available option to minimize this bias. This study also included many children with relatively mild diarrhea and excluded young infants under 6 months of age. This limits comparability of these data to studies including young infants or those only enrolling at admission to hospital. Finally, several pathogens were not sought in the original study, notably enteric viruses and *Aeromonas,* which has been associated with stunting.^44^

At presentation with acute diarrhea, children with a low MUAC were more likely to present with an IMCI danger sign or severe dehydration. This increased risk did not appear to be driven by the presence of high-risk enteric pathogens, but may be related to the social constraints and host vulnerabilities commonly associated with acute malnutrition. Therefore, interventions targeting host factors and social determinants, as opposed to specific pathogens, may be more likely to improve the outcomes of children with malnutrition and diarrhea. In this analysis, being stunted was not associated with diarrhea severity at presentation, as measured by IMCI dangers signs and severe dehydration. Future studies should clarify the role of low HAZ as a risk factor for diarrhea outcomes.

## Supplementary Material

Supplemental Appendix.
