# Supplementary material for: Impact of Childhood Nutritional Status on Pathogen Prevalence and Severity of Acute Diarrhea
Source: Am J Trop Med Hyg. 2017 Aug 21;97(5):1337–44. doi: 10.4269/ajtmh.17-0139 (PMC5817755; doi:10.4269/ajtmh.17-0139)
Supplement: Supplementary file 1 [file tpmd170139.SD1.pdf]

Supplemental Appendix Table 1

Demographic and clinical differences at diarrhea presentation among children with MAM or SAM compared with children without either condition

	SAM (MUAC < 11.5 cm) *n* = 32	MAM (MUAC < 12.5 cm and ≥ 11.5 cm) n = 65	No MAM or SAM (MUAC 12.5 cm) *n* = 1,294
	N/mean (%/SD)	*N*/mean (%/SD)	*N*/mean (%/SD)
Child			
Hospital of presentation			
Homa Bay	21 (65.6)	41 (63.1)	648 (50.0)
Kisii	11 (34.4)	24 (36.9)	646 (50.0)
Age in months	12.9 (8.9)	12.6 (7.4)	24.7 (15.1)
Sex (male)	14 (43.8)	36 (55.4)	692 (53.5)
Currently breast-feeding (if < 24 months)	19 (65.5)	44 (74.6)	542 (78.4)
Months exclusively breastfed	4.7 (1.6)	4.6 (1.8)	5.2 (1.9)
Stunted (HAZ < −2)	15 (51.7)	20 (31.8)	194 (15.4)
Blood in stool	0 (0.0)	0 (0.0)	17 (1.3)
Malaria RDT+	6 (18.8)	6 (9.2)	130 (10.1)
Caregiver			
Biological mother is primary caregiver	30 (93.7)	61 (93.9)	1,210 (93.5)
Caregiver education			
Primary or less	22 (84.6)	40 (72.7)	526 (47.3)
Some secondary	3 (11.5)	10 (18.2)	346 (31.1)
Greater than secondary	1 (3.9)	5 (9.1)	241 (21.7)
Caregiver HIV infected	9 (30.0)	15 (25.4)	112 (9.4)
Socioeconomic status			
Income < 5,000 KSH	21 (65.6)	35 (53.9)	473 (36.7)
Persons per room in house	2.6 (1.6)	2.6 (1.1)	2.3 (1.3)
Improved water source	25 (78.1)	49 (75.4)	1,068 (82.8)
Improved toilet	25 (78.1)	61 (93.9)	1,257 (97.2)
Care seeking			
Previously sought care for this illness	10 (31.3)	28 (43.8)	345 (28.0)
Consulted a traditional healer	2 (6.3)	0 (0.0)	18 (1.4)
> 1 hour travel time to clinic	9 (28.1)	23 (35.4)	206 (15.9)

HAZ = height-for-age z score; HIV = human immunodeficiency virus; MAM = moderate acute malnutrition; MUAC = mid-upper arm circumference; SAM = severe acute malnutrition; SD = standard deviation.

Supplemental Appendix Table 2

Enteric pathogens in children with severe, moderate and no acute malnutrition

	Severe acute malnutrition (MUAC < 11.5 cm) *N* = 32	Moderate acute malnutrition (MUAC ≥ 11.5 < 12.5 cm) *N* = 65	No MAM or SAM (MUAC ≥ 12.5 cm) *N* = 1,293
Infection	*N* (%)	*N* (%)	*N* (%)
Bacteria			
Campylobacter species	2 (6.3)	3 (4.6)	96 (7.4)
EAEC	9 (32.1)	8 (18.6)	111 (13.3)
EIEC	1 (3.6)	1 (2.3)	25 (3.0)
EHEC	0 (0.0)	0 (0.0)	2 (0.2)
EPEC-atypical	1 (6.3)	0 (0.0)	18 (2.3)
EPEC-typical	2 (7.1)	2 (4.7)	32 (3.8)
ETEC	0 (0.0)	2 (4.7)	35 (4.2)
Salmonella species	0 (0.0)	2 (3.1)	16 (1.2)
Shigella species	2 (76.3)	2 (3.1)	61 (4.7)
Protozoa			
Giardia species	0 (0.0)	1 (1.8)	129 (10.7)
Cryptosporidium species	2 (6.7)	1 (1.8)	51 (4.3)
Entaeombea species	1 (3.3)	0 (0.0)	22 (1.9)
Severity			
≥ 1 danger sign	18 (54.3)	34 (53.1)	402 (31.3)
Severe dehydration	10 (31.3)	16 (25.0)	75 (5.8)

EAEC = enteroaggregative *Escherichia coli*; EIEC = enteroinvasive *E.coli*; EHEC = enterohemmorhagic *E.coli*; EPEC = enteropathogenic *E.coli*; ETEC = enterotoxigenic *Escherichiacoli*; MAM = moderate acute malnutrition; MUAC = mid-upper arm circumference; SAM = severe acute malnutrition.
